# Data linkage of Psychiatric and Maternity data to investigate the pregnancy outcomes of women with Non-affective Psychosis in Scotland

**DOI:** 10.23889/ijpds.v1i1.320

**Published:** 2017-04-19

**Authors:** Joanne Given, Elizabeth Nelson, Frances Kane, Helen Dolk, Gillian Robinson

**Affiliations:** Ulster University

## Objectives

To capture a baseline of public attitudes in Northern Ireland towards data linkage and sharing which can then be reassessed at intervals to measure changes in public trust and understanding.

## Approach

The Life and Times Survey is an annual survey of the attitudes of the public of Northern Ireland to a wide range of social issues. Questions within the survey are grouped into modules, the range of which varies from year to year.

In the 2015 survey a module of questions and vignettes was added relating to data sharing. These questions were derived mainly from the ‘Dialogue on Data’ report from ESRC and Ipsos Mori in 2014 and related to the key theme of ‘Public understanding and views of sharing of health data, data linking, and relevant safeguards’.

Each year a systematic random sample of addresses is used to identify selected respondents. Interviews are then carried out face to face in the respondent’s home via Computer Assisted Personal Interviewing. A self-completion questionnaire is also included at the end of the interview.

It is intended that the module relating to public attitudes towards data linkage and sharing are repeated at intervals, possibly every 3 to 5 years, to assess changes in levels of public trust and understanding.

## Results

1200 respondents completed the NILTS between October and December 2015. The dataset is currently being cleaned and will be ready for analysis in April 2016. It is intended that the results of the survey be presented at the IPDLN conference.

## Conclusions

A baseline measure of public attitudes towards data linkage and sharing has been made in Northern Ireland. Repeating this assessment at intervals will make it possible to measure changes in the levels of public trust and understating over time.

